# Photon-counting statistics-based support vector machine with multi-mode photon illumination for quantum imaging

**DOI:** 10.1038/s41598-022-20501-3

**Published:** 2022-10-05

**Authors:** Jin-Woo Kim, Jeong-Sik Cho, Christian Sacarelo, Nur Duwi Fat Fitri, Ju-Seong Hwang, June-Koo Kevin Rhee

**Affiliations:** 1 School of Electrical Engineering, KAIST, Daejeon, 34141 Republic of Korea; 2 QOptics Research Centre, GQT Korea, Seoul, 05854 Republic of Korea

**Keywords:** Optics and photonics, Physics

## Abstract

We propose a photon-counting-statistics-based imaging process for quantum imaging where background photon noise can be distinguished and eliminated by photon mode estimation from the multi-mode Bose–Einstein distribution. Photon-counting statistics show multi-mode behavior in a practical, low-cost single-photon-level quantum imaging system with a short coherence time and a long measurement time interval. Different mode numbers in photon-counting probability distributions from single-photon illumination and background photon noise can be classified by a machine learning technique such as a support vector machine (SVM). The proposed photon-counting statistics-based support vector machine (PSSVM) learns the difference in the photon-counting distribution of each pixel to distinguish between photons from the source and the background photon noise to improve the image quality. We demonstrated quantum imaging of a binary-image object with photon illumination from a spontaneous parametric down-conversion (SPDC) source. The experiment results show that the PSSVM applied quantum image improves a peak signal-to-noise ratio (PSNR) gain of 2.89dB and a structural similarity index measure (SSIM) gain of 27.7% compared to the conventional direct single-photon imaging.

## Introduction

Quantum imaging technology is a single-photon-based technology that improves image quality compared to classical imaging technology by utilizing quantum correlation. These single-photon-based technologies include light detection and ranging (LiDAR), with a high loss rate due to scattering and long-distance transmission, and single-photon emission tomography (SPET), which requires using a single photon for non-destructive imaging of the human body^1–4^. These techniques utilize quantum correlation to achieve a high signal-to-noise ratio (SNR) and image quality improvement even at the single-photon level. Since Seth Lloyd introduced quantum illumination in 2008, technologies such as quantum ghost imaging and heralded quantum imaging have been actively studied^5–10^. Many studies have recently been conducted to improve image quality by post-processing using machine learning on quantum imaging^11,12^. However, the technologies are not capable of eliminating noise itself. Therefore, we thought that the image quality could be improved if some features showed the difference between a signal and noise at the single-photon level.

In quantum imaging, preparing a correlated photon source is essential. An entangled photon-pair or a heralded photon-pair are often used as the photon source^13–21^. These photon sources have temporal and spatial correlations. Spontaneous parametric down-conversion (SPDC) process is most commonly used to prepare a pair of signal and idler photons. It is known that the photon pairs produced by SPDC are in a coherent state and that the photon-counting statistics of these photon pairs follow a Poisson distribution. On the other hand, a quasi-monochromatic thermal photon follows a multi-mode Bose–Einstein (mmBE) distribution^22–31^. Since the background noise, which is a thermal light filtered by a optical bandpass filter, has a short coherence time of in the range of *ps*, while the laser has a long coherence time in the range of *ns*, the signal and noise introduce different physical properties. This introduces different probability desnsity funcitons (pdf) of the signal and noise. The difference in photon-counting statistics for photon sources and noise in quantum imaging suggests a possibility of discovering a new approach to quantum imaging technology by discriminating the laser signal from a quasi-modnochromatic thermal noise. Unfortunately, eliminating noise from the image using the photon count statistics takes much time since the distribution of photons needs to be compared with a mathematical model. This article proposes to use a support vector machine (SVM) to solve this problem for single photon imaging.

To prepare a photon source with quantum correlation, we generated a 1554-nm signal photon and an 809-nm idler photon by injecting a 532-nm pump beam onto the periodically poled lithium niobate (PPLN) crystal. The signal photon is measured by a single-photon detector module (SPDM) after it passes through the telescope traveling in free space to illuminate an object on a mirror at 5 meters away. The experiment includes incandescent light to simulate the background photon noise, a quasi-monochromatic thermal light. The SVM is trained to distinguish the illuminating photon source from the background photon noise and applies it to each pixel to create an image of the object. To determine whether our proposed method is effective, image quality factors, namely, the peak signal-to-noise ratio (PSNR) and the structural similarity index measure (SSIM)^32^, were calculated after the image output was compared with the ground truth. Further, our study showed that quantum statistics-based analysis is effective not only for quantum imaging but also for general single-photon imaging systems.

## Photon-counting statistics based approach

It is known that photon-counting statistics in a coherent state can be modeled as a Poisson distribution. Since a photon-pair generated from SPDC follows the coherent characteristics of the incident pump laser, the coincidence count of the photon-pair can also be modeled with the Poisson distribution^22–27^. On the other hand, the photon counting distribution for only one of the signal or idler photons has a different scenario. When the time interval between measurements is shorter than the coherence time of the photon, the photon-counting statistics of signal (or idler) photons follow the Bose–Einstein distribution^28,29^. In a practical, low-cost experimental environment that does not satisfy this condition, the photon-counting statistics of the signal (or idler) photon become a multi-mode case and follow the mmBE distribution. The probability distribution for the number of photons *n* of the mmBE distribution is expressed as

1 $$\begin{aligned} \begin{aligned} P_m(n) = \frac{\Gamma (m+n)}{n!\Gamma (m)}\frac{m^m{\langle n\rangle }^n}{\left( m+{\langle n\rangle }\right) ^{m+n}}, \end{aligned} \end{aligned}$$

where ${\langle n\rangle }$ is the mean number of photons and *m* is the mode number. Here the *m* can be changed according to the experiment setup. When the photon source has the coherence properties of coherence area $A_c$ and coherence time $T_c$, and the photon measurement setup is constructed with a detection area *A* and measurement time interval *T*, Mandel and Zardecki showed that under the condition of $A\gg A_c$ and $T\gg T_c$, mode number *m* can be approximated as $m=(A/A_c)(T/T_c)$^27,30,31^. A background photon noise from a black body has incoherent characteristics, but when it passes through a spectral filter, the background photon noise becomes a quasi-monochromatic thermal photon noise with partially coherent characteristics^33^. This makes background photon noise follow a mmBE distribution. Interestingly, a Poisson distribution can also be approximated as mmBE distribution with $m=\infty$, which produces all the statistics of the coincidence count of the heralded photon pairs, the count of the signal photons, and the count of the background photon noise changes to have mmBE distributions, but with different mode numbers. The statistical difference of the photons eventually shows that it is possible to distinguish the photon source from the background photon noise utilizing photon-counting statistics.

Two experiments were conducted to obtain the mode numbers of the signal photon and the background photon noise. Generally, studies related to photon count statistics make the measurement time interval shorter than the coherence time so that the observed photon becomes a single mode. In our study, since the mode number of the signal and the background photon noise can be set differently, there is no need to configure an extremely sensitive experimental setup that requires dealing with coherence. In order to obtain experimental data of the background photon noise, we placed an incandescent lamp near the telescope and set the measurement time interval to 25ms.

**Figure 1:**
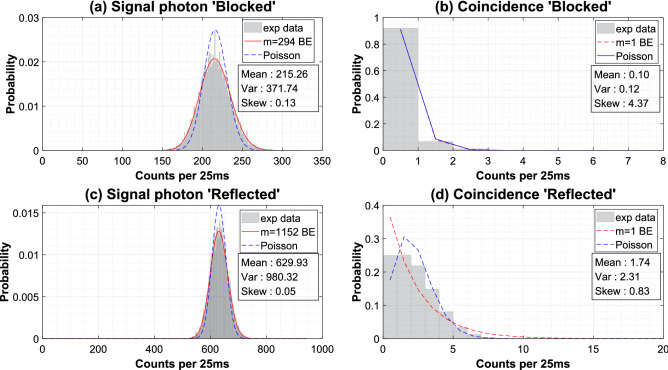
Photon count statistics from the heralded photon-pair generation experiment. Each experiment was performed twice, with and without the background photon noise. (**a**) Signal photons and (**b**) photon-pair coincidence were counted. 10,000 data were collected for each training case with a photon counting time interval of 25 ms. (**c**) The statistics of signal photons is fit to the mmBE distribution and (**d**) the coincidence statistics of photon-pairs is fit to a Poisson distribution.

Figure 1 shows the photon-counting statistics of our experiment, which we fit to mmBE distributions. The fitting of the mathematical modeling to the experimental value based on the point at which the Jensen–Shannon distance is the minimum confirmed that the signal photon was $m=1152$ and the background photon noise was $m=294$, and the resulting value was similar to the prediction. The coincidence count of photon pairs shows a tendency to follow the Poisson distribution, but that of the background photon noise is mixed and has a weak influence from the Bose-Einstein distribution. It is possible to distinguish the signal from the background photon noise by fitting the experimental data to Eq. (1) (see the Supplementary Note 1), but it becomes ambiguous when both signal photon and background noise are mixed when used for quantum imaging. To avoid this time issue, the SVM trained the difference in probability distribution and applied it to quantum imaging.

## Photon classification using SVM algorithm

The SVM is a kind of supervised learning requiring training data consisting of a training vector and a corresponding correct label. In general, machine learning trains the 2D image itself and classifies the images for image processing. This kind of machine learning is known as image classification, and it classifies images with features such as animals, cars, and text^11,12^. However, we propose a photon-counting statistics-based support vector machine (PSSVM) with a completely different application that can be used for quantum and single-photon imaging. We use the PSSVM to distinguish signal photons from the background photon noise utilizing the photon-counting statistics.

Training the PSSVM and classifying the data obtained from experiments requires the pre-processing of data so that the PSSVM can use it. In the pre-processing stage, the data is converted into *k*-dimensional vectors and is normalized to prepare input vectors. The count data $d_i$ is collected *N* times for the measurement time interval of $T=25$ms with a free-running SPDM. First, the entire data set ${\varvec{D}}=\{d_1,d_2,\cdots ,d_N\}$ is reconstructed to *l* number of vectors as

2 $$\begin{aligned} \varvec{X'}_j=\{d_{k(j-1)+1},d_{k(j-1)+2},\cdots ,d_{k(j-1)+k}\}, \qquad \forall \,j=1,2,\cdots ,l, \end{aligned}$$

where the number *l* and dimension *k* satisfy $kl=N$. To minimize the effect of outliers, the data $d_i$ are normalized by the average of corresponding vectors $X'_j$:

3 $$\begin{aligned} x_i=\frac{d_i}{{\langle \varvec{X'}_j\rangle }+\delta }, \qquad \forall d_i\in \varvec{X'}_j\quad \mathrm {and}\quad j=1,2,\cdots ,l, \end{aligned}$$

where ${\langle \varvec{X'}_j\rangle }$ is the average of the vector $\varvec{X'}_j$ and $\delta >0$ is a small positive constant to avoid divide-by-zero errors. The data $x_i$ obtained after normalization are a component of *l* input vectors ${\varvec{X}}_j=\{x_{k(j-1)+1},x_{k(j-1)+2},\cdots ,x_{k(j-1)+k}\}$ for the PSSVM. The input vector ${\varvec{X}}_j$ defined here is a general form regardless of the type of experiment or measurement data, and we will redefine the input vectors for various cases used in experiment analysis.

Among the several types of SVM, we used the $\nu$-SVR model^34^ for this study. When *l* number of ${\varvec{X}}_j\in {\mathbb {R}}^k$ and $z_j\in \{-1,1\}$ is given, a $\nu$-SVR requires that the following optimization problem be solved in

4 $$\begin{aligned} \begin{aligned} \min _{{\varvec{w}},b,\xi ,\xi ^*,\varepsilon }\quad&{\frac{1}{2}{\varvec{w}}^T{\varvec{w}}+C\left( \nu \varepsilon +\frac{1}{l}\sum _{j=1}^{l}{(\xi _j+\xi ^*_j)}\right) },\\ \mathrm {subject\; to}\;\qquad&({\varvec{w}}^T\phi ({{\varvec{X}}_j})+b-z_j\leqslant \varepsilon +\xi _j),\\&z_j-({\varvec{w}}^T\phi ({\varvec{X}}_j)+b\leqslant \varepsilon +\xi ^*_j),\\&\xi _j\xi ^*_j\geqslant 0,\;j=1,\cdots ,l,\;\varepsilon \geqslant 0, \end{aligned} \end{aligned}$$

where the penalty parameter $C>0$, $\varepsilon$ means incentive loss, and the control parameter satisfies $\nu \in (0,1]$. ${\varvec{w}}$ and *b* from the Eq. (4) are components of a hyperplane ${\varvec{w}}^T\phi ({\varvec{X}}_j)+b=0$, where ${\varvec{w}}$ is the normal vector of the hyperplane. $\phi ({\varvec{X}}_j)$ is a kernel function that maps the input vector ${\varvec{X}}_j$ to a higher dimension. In our study, a radial basis kernel is used.

Since the optimization problem form of Eq. (4) is challenging to solve, it is easier to solve the problem by changing it to a dual problem in the form of a Lagrangian primal problem. For Lagrangian multiplier $\varvec{\alpha }$, the dual problem of $\nu$-SVR is given as

5 $$\begin{aligned} \begin{aligned} \min _{\varvec{\alpha },\varvec{\alpha }^*}\qquad&{\frac{1}{2}\left( \varvec{\alpha }-\varvec{\alpha }^*\right) ^TQ\left( \varvec{\alpha }-\varvec{\alpha }^*\right) +{\varvec{z}}^T\left( \varvec{\alpha }-\varvec{\alpha }^*\right) },\\ \mathrm {subject\;to}\qquad&{\varvec{e}}^T\left( \varvec{\alpha }-\varvec{\alpha }^*\right) =0,\quad {\varvec{e}}^T\left( \varvec{\alpha }+\varvec{\alpha }^*\right) \le C\nu ,\\&0\le \alpha _j,\alpha _j^*\le C/l,\quad j=1,2,\ldots ,l, \end{aligned} \end{aligned}$$

where ${\varvec{e}}$ is an *l*-length all-ones vector. An *l* by *l* positive semi-definite matrix *Q* is defined as $Q_{ij}\equiv \phi ({\varvec{X}}_i)^T\phi ({\varvec{X}}_j)$. To distinguish the signal photon and the background photon noise, half of the indicator vector ${\varvec{z}}$ is $z_{j}=1$ and the other half is $z_{j}=-1$. When the testing input vector ${\varvec{X}}\in {\mathbb {R}}^k$ is given, the trained $\nu$-SVR returns the indicator vectors

6 $$\begin{aligned} \begin{aligned} y=\sum _{j=1}^{l}{\left( -\alpha _j+\alpha _j^*\right) \phi \left( {\varvec{X}}_j\right) ^T\phi \left( {\varvec{X}}\right) }. \end{aligned} \end{aligned}$$

In the imaging experiment, the input data ${\varvec{X}}$ at (*u*, *v*) pixel coordinates is given in the form of ${\varvec{X}}_{p,q}(u,v)$, where $p\in \left\{ s,c\right\}$ and $q=1,2,\ldots ,M$. Here, $p=s$ indicates a single-photon imaging experiment using signal photons, and $p=c$ denotes a quantum imaging experiment using the coincidence of the photon pair. Substituting ${\varvec{X}}_{p,q}(u,v)$ into the test vector in Eq. (6), we get $y_{p,q}(u,v)$. An objective can be modeled as

7 $$\begin{aligned} \begin{aligned} O_p(u,v)=\frac{1}{M}\sum _{q=1}^{M}{y_{p,q}(u,v)}. \end{aligned} \end{aligned}$$

Unlike Eq. (7) is treating the information of mode number, the direct imaging model $O_D(u,v)=\frac{1}{lM}\sum _{i=1}^{lM}{d_{c,i}(u,v)}$ or the conventional quantum ghost imaging model $O_G(u,v)=\frac{1}{lM}\sum _{i=1^{lM}{d_{c,i}(u,v)}}$ uses only the average of raw data.

## Experiment

In order to verify the performance of the PSSVM, we demonstrate two quantum imaging experiments. First, as initially intended, quantum imaging was performed with the coincidence count of the photon pairs. In this experiment, the coincidence count followed a Poisson distribution, the same as a mmBE distribution with infinite mode numbers. On the other hand, the background photon noise follows a mmBE distribution with a finite mode number (see the Supplementary Note 2). For this reason, we expected the PSSVM to improve the image quality easily. The second experiment demonstrates single-photon imaging using only signal photon counts of heralded photon pairs. In this case, since both the signal photon and the background photon noise follow mmBE distributions with finite mode numbers, it was not easy to compare the distribution difference rather than the first experiment.

The experimental setup is divided into two layers, an optical layer and a computational layer. In the optical layer, a telescope transmits signal photons and receives the photons reflected from an object. The computational layer contains the PSSVM algorithm and the NIM-BIN logic gates to generate the coincidence count. A photon source of heralded photon pair of a signal photon at 1554 nm and an idler photon at 809 nm was prepared using the uni-directional SPDC experimental setup. In this study, an InGaAs-based SPDM with 20% quantum efficiency was used for signal photons, and a Si-based SPDM with 70% quantum efficiency was used for idler photons. For signal and idler photons, the dark counts were 700 counts per second (cps) and 2000 cps, respectively. Including these dark counts, the signal photons and idler photons showed 3000 cps and 15,000 cps respectively, and the corresponding coincidence count was 200 cps.

**Figure 2:**
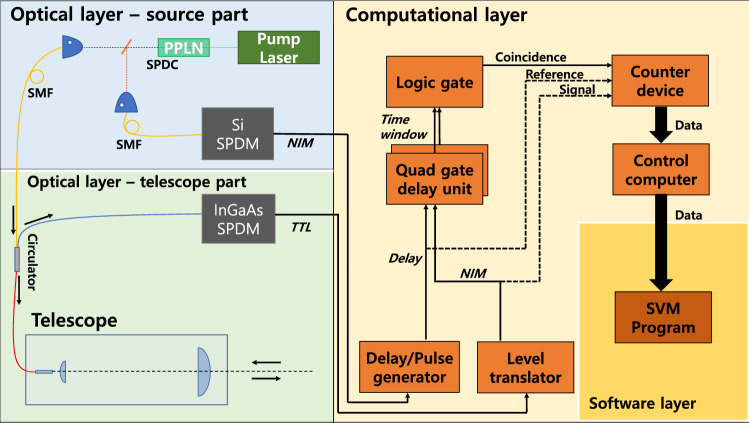
A schematic diagram of the whole experimental set up.

**Figure 3:**
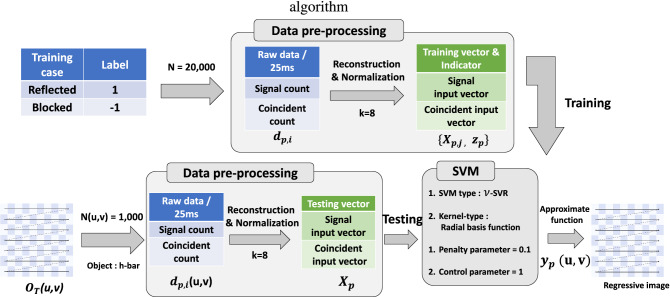
A schematic diagram of the PSSVM algorithm. The PSSVM works as a binary pattern classifier, requiring data of both ‘Reflected’ and ‘Blocked’ types. The training data is prepared as a *k*-dimensional input vector containing information of photon distribution through pre-processing. The trained PSSVM is applied to each of the 2500 pixels of the image to obtain an indicator vector $y_p(u,v)$. In our study, the penalty parameter is $C=0.1$, the control parameter is $\nu =1$, and the kernel function $\phi (\cdot )$ is the radial basis kernel.

In the computational layer, the electrical signals from the InGaAs-based SPDM and Si-based SPDM must be pre-processed so that the counter device can treat signals as shown in Fig. 2. Since the signal photon flies 10m in free space and electrical signals pass through various equipment, the electrical signals from each SPDM do not coincide in time. To compensate for the time difference between electrical signals, we added a delay of 287 ns using a delay/pulse device, and obtained the coincidence count using an AND gate. The counter device generates data with a time bin of 25 ms for the signal photon count and coincidence count. The data is loaded into the computer, pre-processed, and then used as input for the PSSVM.

Figure 3 shows how the PSSVM was trained in the actual experiment and processed the data. The training data used in the single-photon imaging experiment and the quantum imaging experiment are given as $d_{p,i}$ for $i=1,2,\ldots ,N$ and $p\in \left\{ s,c\right\}$. For single-photon imaging experiments ($p=s$) and quantum imaging experiments ($p=c$), the total number of training data in each training sample is N=20,000. Among the 20,000 training data items, half is taken when a mirror reflects signal photons (‘Reflected’ type; $y_i=1$), and the other half is when there is only background photon noise (‘Blocked’ type; $y_i=-1$). The training data undergo reconstruction and normalization to transform into the form of *k*-dimensional input vectors. In obtaining an image from the PSSVM, we prepared 1000 raw data items $d_{p,j}{u,v}$ for each pixel, and transformed those data to *k*-dimension input vectors ${\varvec{X}}_{p,q}(u,v)$. The pre-trained PSSVM produced indicator vector $y_{p,q}(u,v)$ from the input vector, and we reconstructed the object image using Eq. (7). In this study, the trained PSSVM algorithm took about 0.29 milliseconds for each pixel to process the experimental results, while the comparison algorithm using a brute force method took 0.82 s.

**Figure 4:**
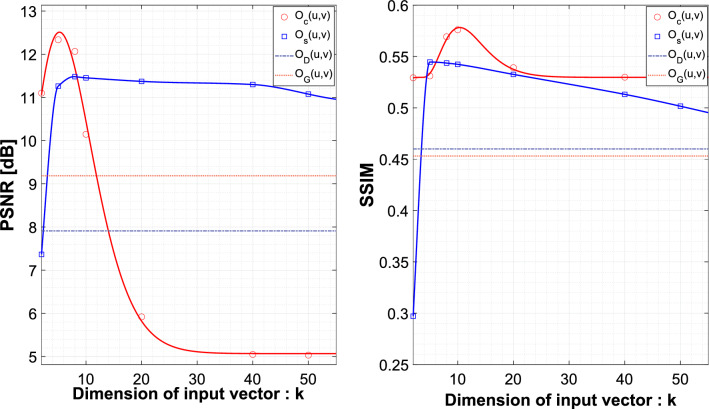
The graphs respectively show the PSNR change and SSIM change according to the size of the input vector. The experiment is demonstrated for 7 dimensions: $\left\{ 2,5,8,10,20,40,50\right\}$. As a comparison group, single-photon imaging $O_D(u,v)$ and quantum imaging $O_G(u,v)$ are indicated repectively by blue and red dashed lines. The single-photon imaging $O_s(u,v)$ and quantum imaging $O_c(u,v)$ with PSSVM applied to each are respectively indicated by blue squares and red circles.

We calculated the PSNR and SSIM of the obtained images to confirm the image quality. Since the size of the input vector is an essential factor for the PSSVM, the results were analyzed by changing the *k* value. Fig. 4 shows that the PSSVM applied quantum images have high PSNR and SSIM for optimized $k=8$.

**Figure 5:**
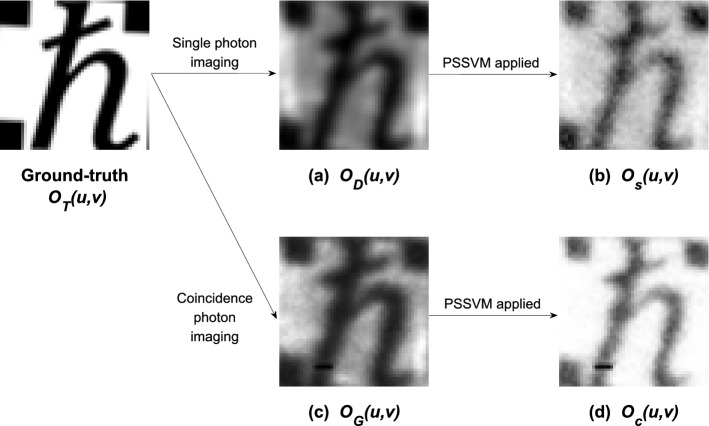
50 $\times$ 50 pixel ground-truth image $O_T(u,v)$. (**a**) Single-photon image $O_D(u,v)$. (**b**) PSSVM applied single-photon image $O_s(u,v)$. (**c**) Quantum image $O_G(u, v)$. (**d**) PSSVM applied quantum image $O_c(u,v)$.

**Table 1:** Performance of image generation using raw data. Labels (a–d) correspond to those in Fig. 5.

Single photon imaging		Coincidence photon imaging	
Raw data image	PSSVM applied	Raw data image	PSSVM applied
(a) $O_D(u,v)$ PSNR = 7.91 dB SSIM = 0.46	(b) $O_s(u,v)$ PSNR = 11.48 dB SSIM = 0.54	(c) $O_G(u,v)$ PSNR = 9.18dB SSIM = 0.45	(d) $O_c(u,v)$ PSNR = 12.07 dB SSIM = 0.57

Within the optimized *k* condition, the direct imaging model $O_D(u,v)$ has image qualities PSNR = 7.91 dB and SSIM = 0.46, and the conventional quantum ghost imaging model $O_G(u,v)$ has PSNR = 9.18 dB and SSIM = 0.45. In our experiment, the quantum ghost image achieved better results than the direct image, as seen in previous studies. Compared with the ground-truth image $O_T(u,v)$, the $O_s(u,v)$ has PSNR = 11.48dB and SSIM = 0.54, and $O_c(u,v)$ has PSNR = 12.07 dB and SSIM = 0.57. Figure 5 shows the four images in grayscale, and Table 1 summarizes the information of each image’s quality factors. By applying the PSSVM, the single-photon imaging shows PSNR gain of 3.57 dB and quantum imaging shows PSNR gain of 2.89dB. The PSSVM applied single-photon imaging $O_s(u,v)$ shows a PSNR gain of 2.3 dB higher than the conventional quantum imaging $O_G(u,v)$.

## Discussion

Photon-counting statistics of the photon source and the background photon noise can be modeled as a multi-mode Bose–Einstein distributions, importantly with different mode numbers, for different photon sources manifest different coherence signatures. We demonstrate that mode numbers of the photon source for illumination and the background photon noise are significantly different, and we verify that the experimental results coincide with the theoretical predictions. This suggests an intuition that the photon-counting statistics can be used in image processing. However, the mode number estimation for every pixel by fitting the experimental data to the theoretical model requires too much time cost. In order for a practical realization, we used the SVM to reduce the processing time. The experiment was conducted to take images of a 6 $\times$ 6-mm object at a distance of 5 m with a 50 $\times$ 50 image pixels, including an incandescent light source for a background photon noise. The result of the experiment confirmed that the PSSVM improved image quality factors in both single-photon imaging and heralded photon-pair imaging. Interestingly, single-photon imaging using the PSSVM shows better image qualities than conventional quantum imaging. Our experimental investigation suggests that similar or better image quality can be obtained with a single-photon imaging setup without enhancement by photon heralding that requires much more image acquisition time and much higher system complexity. However, even though it is far better than coincidence counting for heralded photon pairs, our proposed scheme still requires photon count distribution information with large enough counts of photons per each pixel. This issue can be resolved in future works with faster single photon detectors that can be used with high power laser sources. In this study, since the signal classification is expected to be achieved only by the number of modes related to coherence properties, the performance of the proposed method seems to be independent from SNR. As a future work, experimental investigation is anticipated to see how much PSSVM is affected by SNR in the small SNR regime.

## Methods

**Figure 6:**
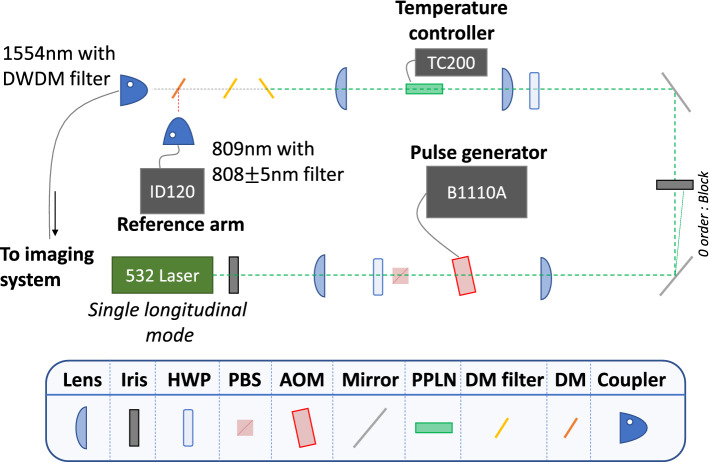
Schematic diagram of heralded photon-pair generation. Pairs of 1554-nm signal photon and 809-nm idler photon are generated by SPDC using a 532-nm pump laser (MSL-F-532; CNI). Two dichroic mirrors remove the pump laser, and each photon is filtered with DWDM ITU Ch29 and 808 ± 5 nm narrow bandpass filters. *Iris* Iris pinhole, *HWP* half-wave plate, *PBS* polarization beam splitter, *AOM* acoustooptic modulator, *PPLN* periodically poled lithium niobate crystal, *DM* dichroic mirror.

The heralded photon-pair is prepared by SPDC experimental setup as shown in Fig. 6. Since the C-band wavelength has a low attenuation rate in air and is safe for human eyes, 1554 nm was selected for the signal photon wavelength. The other photon of the heralded photon-pair, the idler photon has an 809-nm wavelength which has a high detection efficiency for a Si-based single-photon detector. To satisfy the quasi-phase matching condition, a nonlinear optical crystal PPLN with a 7.10-$\upmu$m poling period length was temperature controlled at 187.1$^\circ$C. The pump beam has a center wavelength of 532 nm and a linewidth of 10 MHz. The pump beam was modulated to have a pulse width of 50 ns at a repetition rate of 500 kHz using an acousto-optic modulator (AOM). A dense wavelength division multiplex (DWDM) with a bandwidth of 100 GHz is used to limit the spectrum width of signal photons.

The heralded photon pair passe through the single-mode fiber (SMF) to the telescopic imaging setup to take a picture of the object. The object is printed on polyvinyl chloride (PVC) film of 6 $\times$ 6 mm and pasted on a mirror. The object is a binary image, the black areas are blocked by ink, and the white areas allow photons to be reflected off the mirror through a transparent PVC film. The signal photon propagates through a free space of 5 m, returns to the telescope, passes through a circulator and a DWDM bandpass filter, and is detected with an InGaAs-based SPDM. In our experimental setup, the total loss for signal photons was 3.4 dB. On the other hand, the idler photon passes through only a bandpass filter with a 10 nm bandwidth and is directly measured with the Si-based SPDM. The incandescent lamp is located 22 cm next to the telescope to generate a background photon noise.

The optical layer includes InGaAs SPDM(ID221; IDQ) and Si SPDM(ID120; IDQ). The system layer contains delay/pulse generator(Model DG535; Stanford research systems), Quad gate delay units(Model 794; ORTEC), and a counter device(PCIE-6602; National instruments).

## Supplementary Information

Supplementary Information 1.

Supplementary Information 2.

Supplementary Information 3.

## Data Availability

All data generated or analyzed during this study are included in this published article (and its supplementary information files).
