# Supplementary figures and images for: Photon-counting statistics-based support vector machine with multi-mode photon illumination for quantum imaging
Source: Sci Rep. 2022 Oct 5;12:16594. doi: 10.1038/s41598-022-20501-3 (PMC9534992; doi:10.1038/s41598-022-20501-3)

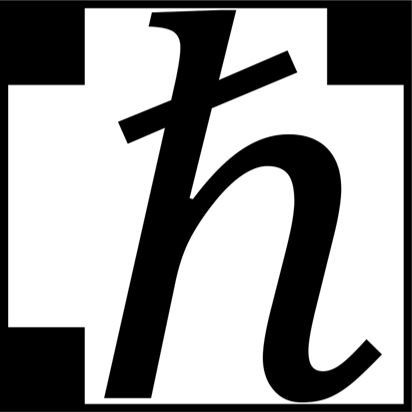

Supplement: Supplementary file 1 — Supplementary Information 1. [file 41598_2022_20501_MOESM1_ESM.zip › GroundTruthImage_raw.png]
