# Supplementary material for: Photon-counting statistics-based support vector machine with multi-mode photon illumination for quantum imaging
Source: Sci Rep. 2022 Oct 5;12:16594. doi: 10.1038/s41598-022-20501-3 (PMC9534992; doi:10.1038/s41598-022-20501-3)
Supplement: Supplementary file 2 — Supplementary Information 2. [file 41598_2022_20501_MOESM2_ESM.pdf]

# Supplementary Note for Photon counting statistics based Support Vector Machine for quantum imaging

**Jin-Woo Kim[1], Jeong-Sik Cho[2], Christian Sacarelo[1], Nur Duwi Fat Fitri[1], Ju-Seong Hwang[1], and June-Koo Kevin Rhee[1,✉]**

[1]School of Electrical Engineering, KAIST, Daejeon, 34141, Republic of Korea
[2]QOptics Research Centre, gqt korea, B-dong 1519, 201 Songpa-daero, Songpa-gu, Seoul, 05854, Repulic of Korea
✉email: rhee.jk@kaist.edu

## Supplementary Note 1: Distribution distance among the multi-mode values

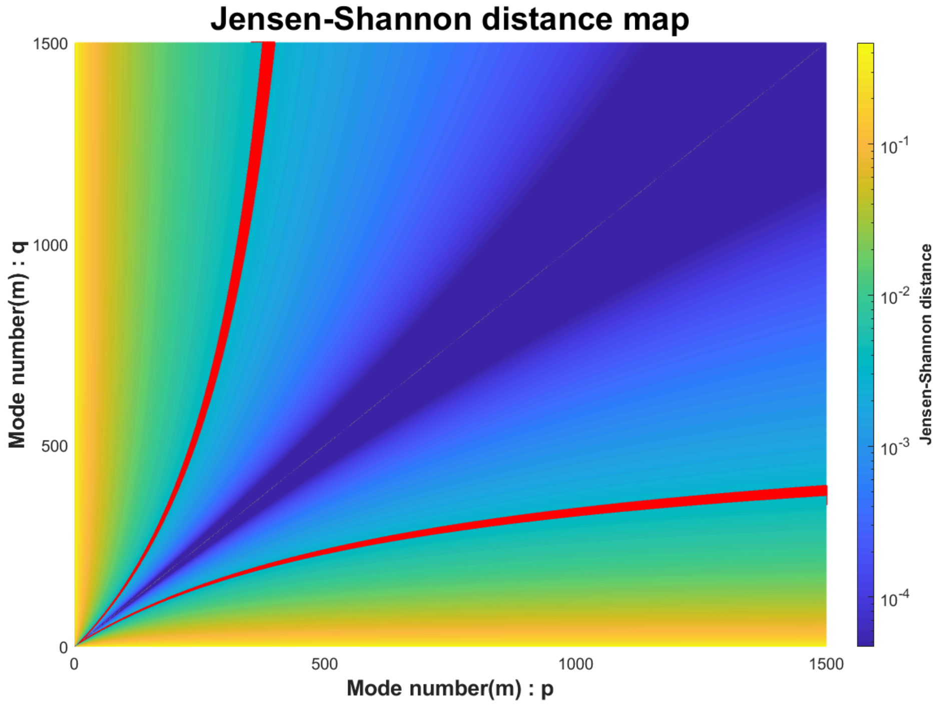


**Supplementary Figure. 1.** The Jensen-Shannon distance (JSD) map for two other mmBE distributions, which have different multi-mode numbers. The red line shows same JSD cases with our experiment.

This study shows that even if photon-counting statistics of both signal photon and background photon noise follows multi-mode Bose-Einstein distribution (mmBE), they can be distinguished if t he multi-mode values are different. However, there are only experimental attempts to distinguish those statistics for m=1152 and m=294, and the reference range for discrimination was not revealed. Supplementary Fig. 1 visualizes the Jensen-Shannon distance (JSD) according to the multi-mode values for the mmBE distributions through simulation and predicts the range of multi-mode values in which our research can operate correctly through the experimental results.

In order to check how two mmBE distributions are different, we calculated the distribution distance between those mmBE distributions using JSD[1]:

$$JSD(P|Q) = \frac{1}{2}D_{KL}(P|M) + \frac{1}{2}D_{KL}(Q|M), \tag{1}$$

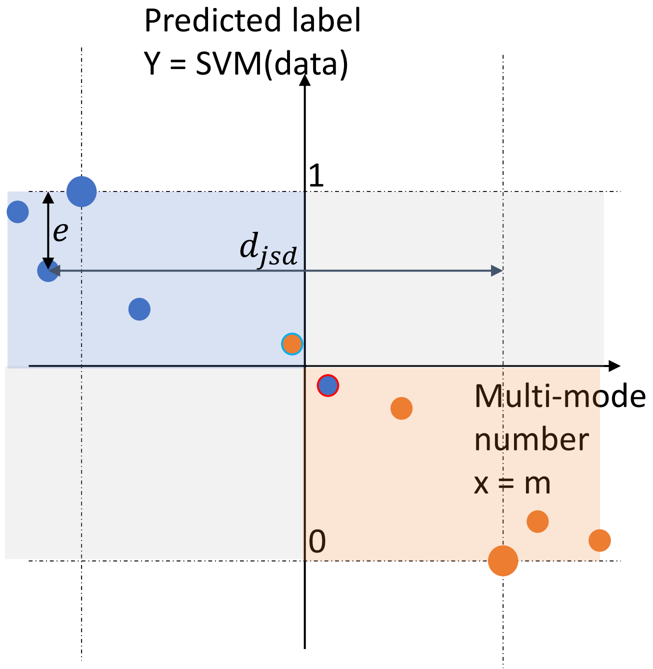


**Supplementary Figure. 2.** Schematic concept diagram to define the Jensen-Shannon distance $d_{jsd}$ from opposite-case training set and the error of PSSVM result $e$. PSSVM is designed to classify 'Reflected' and 'Blocked' cases by learning the distribution difference caused by multi-mode values. The blue area is the case of 'Reflected', where the PSSVM result has a label of 1; the orange area is the case of 'Blocked', where the PSSVM result has a label of 0.

where $M = \frac{1}{2}(P+Q)$ and

$$D_{KL}(P|Q) = \sum_{x \in \chi} P(x) log\left(\frac{P(x)}{Q(x)}\right), \tag{2}$$

for two probability distributions $P(x)$ and $Q(x)$. We evaluate the JSD map for multi-mode values between 0 and 1500 for both $P$ and $Q$. In our experiment, multi-mode values are $m = 1152$ and $m = 294$ for signal photons and background noise photons, respectively. Within this condition the JSD shows $3.6 \times 10^{-3}$, as the red line of Supplementary Fig. 1. This suggests that in the limit of low JSD, our proposed method works properly.

## Supplementary Note 2: Various experiment environment for PSSVM

The quantum imaging in this paper used an incandescent lamp to introduce a background photon noise, located at 22 cm away from the telescope. The PSSVM was trained to construct image against this photon noise. However, it is desired to confirm that it works for other background photon noises with various multi-mode values. We conducted an experiment to investigate how the PSSVM error changes by different multi-mode values corresponding to the different positions of the incandescent lamp.

For this test, the location of the lamp was moved to $\{10\,\text{cm}, 15\,\text{cm}, 30\,\text{cm}, 2\,\text{m}\}$ after training at 22 cm. Data were collected for 'Reflected' and 'Blocked' cases. In order to make the effect of the lamp position generalized, we estimated the JSDs of the photon statistics at these lamp locations with respect to that of the lamp location at 22 cm. The error rates of PSSVM with respect to the JSD are shown in Supplementary Fig. 2, for cases of 'Reflected' and 'Blocked'. As $d_{jsd}$ defined in Supplementary Fig. 2 increases, the error rates of PSSVM $e$ tend to decrease.

Supplementary Fig. 3 shows the error analyses for the cases of 'Reflected' and 'Blocked'. In the figure, the 'Reflected' case shows that the error decreases sharply as $d_{jsd}$ increases, and the coincidence data is more sensitive than the signal data. In the case of 'Blocked', $d_{jsd}$ shows little difference in error, but the signal data has much larger

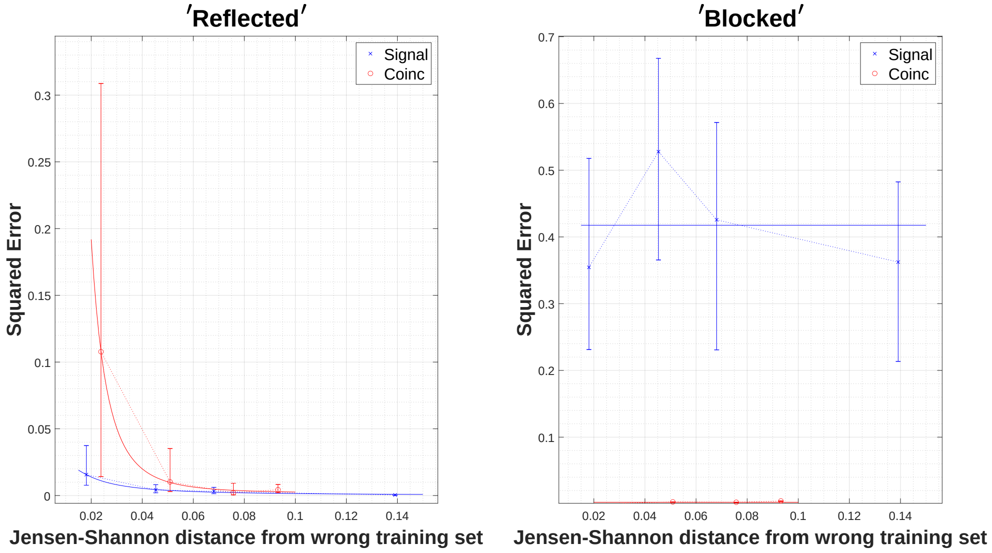


**Supplementary Figure. 3.** Performance analysis of the errors for trained PSSVM for various multi-mode numbers represented by JSD.

error than coincidence data. This leads to a significant improvement in quantum image quality when coincidence photon imaging is used rather than single photon imaging. A mathematical analysis of these phenomena is anticipated to show the possibility of expanding the engineering application of photon statistics as a future work.

## Supplementary Note 3: Raw data list

**1. PSSVM_raw_data.zip : Raw data set for the article**

(1) GroundTruthImage_raw.png : Ground-truth raw image of object in the article. Since the taken image of experiment is rotated and has different scale, we construct the ground-truth image using the GroundTruthImage_raw.png.
(2) Learning_Blocked.csv : Training data for 'Blocked' case without background photon noise.
(3) Learning_Blocked_with_lamp.csv : Training data for 'Blocked' case with background photon noise.
(4) Learning_Reflected.csv : Training data for 'Reflected' case without background photon noise.
(5) Learning_Reflected_with_lamp.csv : Training data for 'Reflected' case with background photon noise.
(6) Imaging_test_data.csv : Test data for PSSVM. This is the raw data of Figure 6. of the article. First and second column are including the position of pixel. The third column is the signal photon counting. The fifth column is the coincidence photon counting. The fourth column is idler photon counting, but we did not use this information for analyzing.

**2. Suppl_raw_data.zip : Raw data set for the supplementary note**

(1) Suppl_testdata_10_Blocked.csv : Test data for 'Blocked' case where the lamp is located at 10 cm away from the telescope.
(2) Suppl_testdata_10_Reflected.csv : Test data for 'Reflected' case where the lamp is located at 10 cm away from the telescope.
(3) Suppl_testdata_15_Blocked.csv : Test data for 'Blocked' case where the lamp is located at 15 cm away from the telescope.
(4) Suppl_testdata_15_Blocked.csv : Test data for 'Reflected' case where the lamp is located at 15 cm away from the telescope.
(5) Suppl_testdata_30_Blocked.csv : Test data for 'Blocked' case where the lamp is located at 30 cm away from the telescope.
(6) Suppl_testdata_30_Reflected.csv : Test data for 'Reflected' case where the lamp is located at 30 cm away from

the telescope.
(7) Suppl_testdata_200_Blocked.csv : Test data for ’Blocked’ case where the lamp is located at 2 m away from the telescope.
(8) Suppl_testdata_200_Reflected.csv : Test data for ’Reflected’ case where the lamp is located at 2 m away from the telescope.
(9) Suppl_traindata_22_Blocked.csv : Training data for ’Blocked’ case where the lamp is located at 22 cm away from the telescope.
(10) Suppl_traindata_22_Reflected.csv : Training data for ’Reflected’ case where the lamp is located at 22 cm away from the telescope.

## References


**1.** Endres, D. M. & Schindelin, J. E. A new metric for probability distributions. *IEEE Transactions on Inf. theory* **49**, 1858–1860 (2003).
